# Adjusting for geographic variation in observational comparative effectiveness studies: a case study of antipsychotics using state Medicaid data

**DOI:** 10.1186/1472-6963-14-355

**Published:** 2014-08-27

**Authors:** Elisabeth Dowling Root, Deborah SK Thomas, Elizabeth J Campagna, Elaine H Morrato

**Affiliations:** Department of Geography and Institute for Behavioral Science, University of Colorado at Boulder, Boulder, CO 80309 USA; Department of Geography and Environmental Sciences, University of Colorado Denver, Denver, CO USA; Colorado School of Public Health, University of Colorado Denver, Anschutz Medical Campus, Aurora, CO USA; Colorado Health Outcomes Program, University of Colorado Denver, Anschutz Medical Campus, Aurora, CO USA

**Keywords:** Comparative effectiveness research, Antipsychotics, Mental health, Small area variation, Geographic information systems, Spatial data analysis, Multilevel modeling

## Abstract

**Background:**

Area-level variation in treatment and outcomes may be a potential source of confounding bias in observational comparative effectiveness studies. This paper demonstrates how to use exploratory spatial data analysis (ESDA) and spatial statistical methods to investigate and control for these potential biases. The case presented compares the effectiveness of two antipsychotic treatment strategies: oral second-generation antipsychotics (SGAs) vs. long-acting paliperiodone palmitate (PP).

**Methods:**

A new-start cohort study was conducted analyzing patient-level administrative claims data (8/1/2008–4/30/2011) from Missouri Medicaid. ESDA techniques were used to examine spatial patterns of antipsychotic prescriptions and outcomes (hospitalization and emergency department (ED) visits). Likelihood of mental health-related outcomes were compared between patients starting PP (N = 295) and oral SGAs (N = 8,626) using multilevel logistic regression models adjusting for patient composition (demographic and clinical factors) and geographic region.

**Results:**

ESDA indicated significant spatial variation in antipsychotic prescription patterns and moderate variation in hospitalization and ED visits thereby indicating possible confounding by geography. In the multilevel models for this antipsychotic case example, patient composition represented a stronger source of confounding than geographic context.

**Conclusion:**

Because geographic variation in health care delivery is ubiquitous, it could be a comparative effectiveness research (CER) best practice to test for possible geographic confounding in observational data. Though the magnitude of the area-level geography effects were small in this case, they were still statistically significant and should therefore be examined as part of this observational CER study. More research is needed to better estimate the range of confounding due to geography across different types of observational comparative effectiveness studies and healthcare utilization outcomes.

**Electronic supplementary material:**

The online version of this article (doi:10.1186/1472-6963-14-355) contains supplementary material, which is available to authorized users.

## Background

Tremendous progress in codifying best practices for conducting comparative effectiveness research (CER) using observational datasets has been made. The Agency for Healthcare Research and Quality (AHRQ) [1] and Patient Centered Outcomes Research Institute (PCORI) [2] have published detailed protocols outlining standards for the design and implementation of CER studies. These reports stress that observational CER studies require multidimensional analytic strategies and careful consideration of which multivariate modeling approaches best represent the structure of the data, the study outcome, and exposure/treatment being studied. What is largely absent from the CER literature is an explicit discussion of how geographical context can affect observational studies and, in particular, introduce bias into statistical analyses (see, for example, the August 2013 special supplement on methods for CER/PCOR in the *Journal of Clinical Epidemiology*). Geographical variation in populations, physician practice patterns, and the availability and accessibility of hospital, specialty, and primary care can influence both treatment choices and care seeking behaviors [3–6]. These differences may, in turn, be reflected in health-care utilization databases often used in CER studies [7]. Spatial statistical methods, which examine small-area variation in these factors, can provide additional information to assist in further alleviating bias in observational CER studies.

The *Dartmouth Atlas of Healthcare* series [8–10] and the *Atlas of U.S. Mortality* [11] provide strong empirical evidence of geographical patterns of mortality, disease, health care resource availability and service utilization across the U.S. Since their publication in the mid-1990’s, numerous studies have demonstrated geographical differences in health and health care for many outcomes and procedures. This literature suggests that two geographic principles govern spatial patterns of care. First, people with similar demographic characteristics and risk factors tend to live near each other, producing larger areal patterns of morbidity and mortality [12]. This is often referred to as the compositional effect, since the geographic patterns are due to the underling composition of the population. These patterns, for a variety of socio-demographic reasons, often translate to variations in service use, particularly hospitalization and emergency department (ED) visits. For example, a county with more low income individuals may exhibit higher rates of ED visits for asthma because the condition is not being managed well due to lack of primary care. These patterns are particularly important for observational CER studies, which rely on electronic medical records, insurance claims or hospital discharge data to measure outcomes. Many of the analytic strategies suggested by AHRQ and PCORI attempt to account for this variation by controlling for, (e.g., multivariate models) or matching on (e.g., propensity scores), patient-level characteristics. If the area-level differences are indeed due to compositional effects, these techniques should be effective in controlling for bias.

The second geographic principle which drives spatial patterns of morbidity and utilization is the notion of areal contextual differences. The care a person receives depends upon the supply of resources available and physician practice patterns in the place the person lives [13]. Recent CER work has introduced instrumental variables (IVs) to capture geographic variation in practice patterns which may affect treatment exposure [14–16]. Unfortunately, one condition of an IV is that it: “is a cause of treatment but has no causal association with the outcome other than through its effect on treatment” [1], which is difficult for many CER studies. The social epidemiology and sociology literature also suggests that health outcomes, service utilization and treatment decisions are affected by social and cultural norms, social ties and neighborhood disorganization, often measured using area-level socioeconomic status [17–21]. The current literature on geographic variation in health has shifted from descriptive analysis of spatial patterns to sophisticated statistical analyses of the contextual factors which contribute to these patterns [22–26]. Contextual factors are ubiquitous in social epidemiological analyses but rarely considered in CER. IVs cannot be used to control for area-level socio-economic or cultural patterns because these variables are often highly related to the outcome under study. As such, there is a need to integrate valuable knowledge from geography, social epidemiology and sociology into CER best practices in order to explore the effect of geographical variations in area-level socioeconomic status (SES) and utilization on both the treatment exposure and the outcome under study.

In this study, we seek to build upon the strong analytic foundation laid by AHRQ and PCORI by suggesting that exploratory spatial data analysis (ESDA) and spatial statistical methods be integrated into CER studies and included in guidelines for rigorous CER methods. This study is based on two hypotheses related to two potential sources of confounding bias: 1) drug prescription patterns (our study exposure) may vary geographically as adoption may be uneven throughout the state, and 2) hospitalization and ED visits (our study outcomes) may vary geographically depending upon local referral and treatment practices and availability of health services (e.g., in urban vs. rural areas). Therefore, we investigate whether area-level geographic variation in treatment and outcomes is a source of confounding bias in this observational comparative effectiveness study. ESDA is a powerful tool which can be used to understand the potential biases produced by area-level compositional and contextual factors in CER studies, while spatial statistical methods can control for these biases. We demonstrate these techniques, and their utility, with a case study that compares the effectiveness of paliperidone palmitate, a long-acting second-generation antipsychotic (SGA), with orally-administered SGA’s among Medicaid patients in the State of Missouri. If area-level variation is a source of confounding bias, it should be investigated as part of good comparative effectiveness research practices.

## Methods

This is a secondary analysis of a retrospective observational study using a new user cohort design. Although we have examined these data using other methods appropriate for observational data (e.g., propensity score matching and provider and patient restricted cohorts), we do not present these analyses here. Rather, we focus on additional geographic analyses not typically included in observational CER studies which aim to determine how geographical differences in the treatment and outcome may affect results. First, we conducted ESDA by: 1) mapping drug utilization, and hospitalization/ED visit rates, 2) smoothing those rates using Spatial Empirical Bayes (SEB) methodologies, and 3) analyzing spatial patterns using Moran’s I and local indicators of spatial association to look for local clustering. Second, we developed multilevel logistic regression models and mapped random effects to evaluate model performance and the effect of spatial differences in utilization and admission rates on the interpretation of our results.

This study received approval from the University of Colorado Denver Multiple Institutional Review Board (IRB) and adhered to Data Use Agreements specified by the State of Missouri.

### Data

Patient-level data were extracted from Missouri MOHealthNet (Medicaid) and Department of Mental Health administrative databases (see Additional file 1: Figure S1 for CONSORT diagram of study population). Medicaid clients with a prescription claim for paliperidone palmitate or an oral SGA agent between August 1, 2009 and April 30, 2010 (inclusive) were identified. Oral SGAs studied were aripiprazole, asenapine, clozapine, iloperidone, lurasidone, olanzapine, quetiapine, risperidone, and ziprasidone. Paliperidone palmitate received FDA approval July 2009, defining the beginning of patient accrual. The most current data available at the time the investigation began (April 30, 2011) was used to define the end of 12-month follow-up observation. Medicaid clients who were Medicare eligible or had Medicaid eligibility less than 12 months before and after the first qualifying pharmacy (Index) claim date were excluded because claims data or observation follow-up would be incomplete. The primary analysis compared outcomes in the New Start Cohorts of paliperidone palmitate (N = 296) and oral SGA (N = 8,675) users.

Mental-health related hospitalization and ED visits in the year post drug initiation were the primary outcomes studied. An outcome was defined as mental health related if the first diagnosis of the claim record was for a mental disorder specified in the AHRQ Mental Health and Substance Abuse Classification [27].

Several baseline (past year) demographic and clinical characteristics were identified as possible confounders. Demographic characteristics included: age, sex, and race. Other patient characteristics included: mental health conditions (identified using ICD-9-CM diagnosis codes), cardiometabolic co-morbidities (diabetes, disorders of lipid metabolism, hypertension, and diseases of the heart, identified using ICD-9-CM codes), frequency of hospitalizations and outpatient visits, receipt of care at a Community Mental Health Center (CMHC), and evidence of case management. Information on the patient and clinical factors used to adjust models is available from prior published studies [28, 29].

Geographic location of the patient was identified using both county and 5-digit zip code of residence. Zip codes were used to categorize patients into state-defined mental health service areas (MHSA). Fifty records were lost due to the inability to assign a geographic location, reducing the sample to 8,921. Study participants were nested into 115 counties (114 counties and 1 independent city) and 27 MHSAs.

### Statistical analysis

#### Spatial empirical bayes smoothing

Rate smoothing is a technique used to: 1) stabilize rates based on small numbers and 2) reduce noise in rates caused by different population sizes [30, 31]. In the context of CER, especially with new drug introductions where study populations are smaller, rate smoothing allows us to examine data at a finer spatial resolution (e.g., zip codes) rather than aggregating data to larger regions (e.g., counties). Rate smoothing increases the ability to discern systematic patterns in the spatial variation of the outcome under study by reducing noise and making trends and patterns more obvious.

Spatial Empirical Bayes (SEB) smoothing was used to create rate maps of paliperidone palmitate and oral SGA utilization and hospitalization and ED visits. SEB averages the rate in each zip code with the rates from neighboring zip codes. SEB smoothing “borrows” data on events from nearby regions (“neighbors”) to stabilize local estimates by weighting the rate based on those neighboring values [32]. For all spatial analyses in this study, neighbors are defined using queen’s case continuity, which creates links between all neighbors sharing a common point on their boundaries. This smoothing method is commonly used with data that is already aggregated to areas, because it uses contiguity to define neighbors. Other methods, such as kernel density smoothing are often typically used, but more often with point-level data where distance between individual events can be measured precisely.

#### Moran’s I and LISA

Smoothed rate maps do not indicate if observable spatial patterns are statistically significant. Moran’s I and local indicators of spatial association (LISA) are two exploratory statistical techniques which test whether or not rates cluster in space and highlight the location of local pockets of mutually similar deviations from the overall mean regional rate.

The Moran's I statistic measures global clustering or, more formally, spatial autocorrelation among rates across all zip codes in the study area [33]. Moran's I is a weighted correlation coefficient, similar to Pearson’s correlation coefficient, where weights are based on spatial proximity of zip codes. Values of Moran’s I range from 1 to -1 and a large positive or negative Moran’s I indicates utilization or admission rates in nearby areas are highly similar (or dissimilar), whereas when Moran's I near 0 indicates no spatial association in the data.

Moran's I is a global test and does not indicate *where* clusters are located in the study area. The LISA was therefore used as an indicator of local spatial association [34]. The LISA measures whether, for each zip code, the utilization or admission rate is closer to the values of its neighbors or to the state average. Significance was assessed using Monte Carlo simulations with 999 permutations. LISA results were then mapped. Significant spatial associations are designated as: 1) ‘high–high’ which indicates that high rates are next to each other (positive spatial autocorrelation), 2) ‘low–high’ indicates that low rates are adjacent to high rates (negative spatial autocorrelation), 3) ‘low–low’ indicates clustering of low rates (positive spatial autocorrelation), 4) ‘high–low’ indicates that high rates are adjacent to low rates (negative spatial autocorrelation), or 5) ‘not significant’ indicates that there is no spatial autocorrelation. SEB rates, Moran’s I and LISA statistics were calculated using OpenGeoDa v1.0.1 and R v2.13.0.

#### Multilevel modeling

After examining the results of the ESDA, we hypothesized spatial patterns of drug utilization and outcomes were clustered within both counties and MHSA. Multilevel logistic regression models where patients were nested within either counties or MHSAs [35, 36] were performed to determine the association between treatment with paliperidone palmitate vs. oral SGA and the likelihood of a mental health hospitalization or ED visit. To determine the extent to which counties/MHSAs were independently associated with outcomes, we used a random intercept model (unadjusted models) to partition the variance between MHSAs and patient-level characteristics. Individual level characteristics (adjusted models) were then added as fixed effects to the model and finally MHSA- or county-level random effects (adjusted random effects models) were included. We used a spatially structured level-2 error term in order to control for potential spatial autocorrelation. Covariance matrices were based on an exponential distance decay function of the form *σ*^2^*exp*{-*d*_*ij*_/*θ*}, such that zip codes close to one another would have a larger covariance estimate than those further apart. As a robustness check, both spatially structured and non-spatially structured random effect models were examined, and estimates were nearly identical. We chose to present the spatially structured error terms because we believe they were more theoretically grounded.

Several iterations of the models were run including: intercept-only random effects, slope-only random effects and both intercept and slope random effects. The intercept-only models were chosen as the “best model” based on model fit statistics and significance tests of the random effects. The intra-class correlation (ICC), which is the ratio of the between group variance to the total variance, was calculated to estimate the percent of variance in the outcome that is between MHSAs or counties. MHSA and county random effects were mapped to examine where the probability of a hospitalization or ED visit deviated significantly from the global mean after controlling for individual-level factors. Regression models were run using the GLIMMIX procedure in SAS v9.3. No adjustment was made for multiplicity.

## Results and discussion

**Figure 1:**
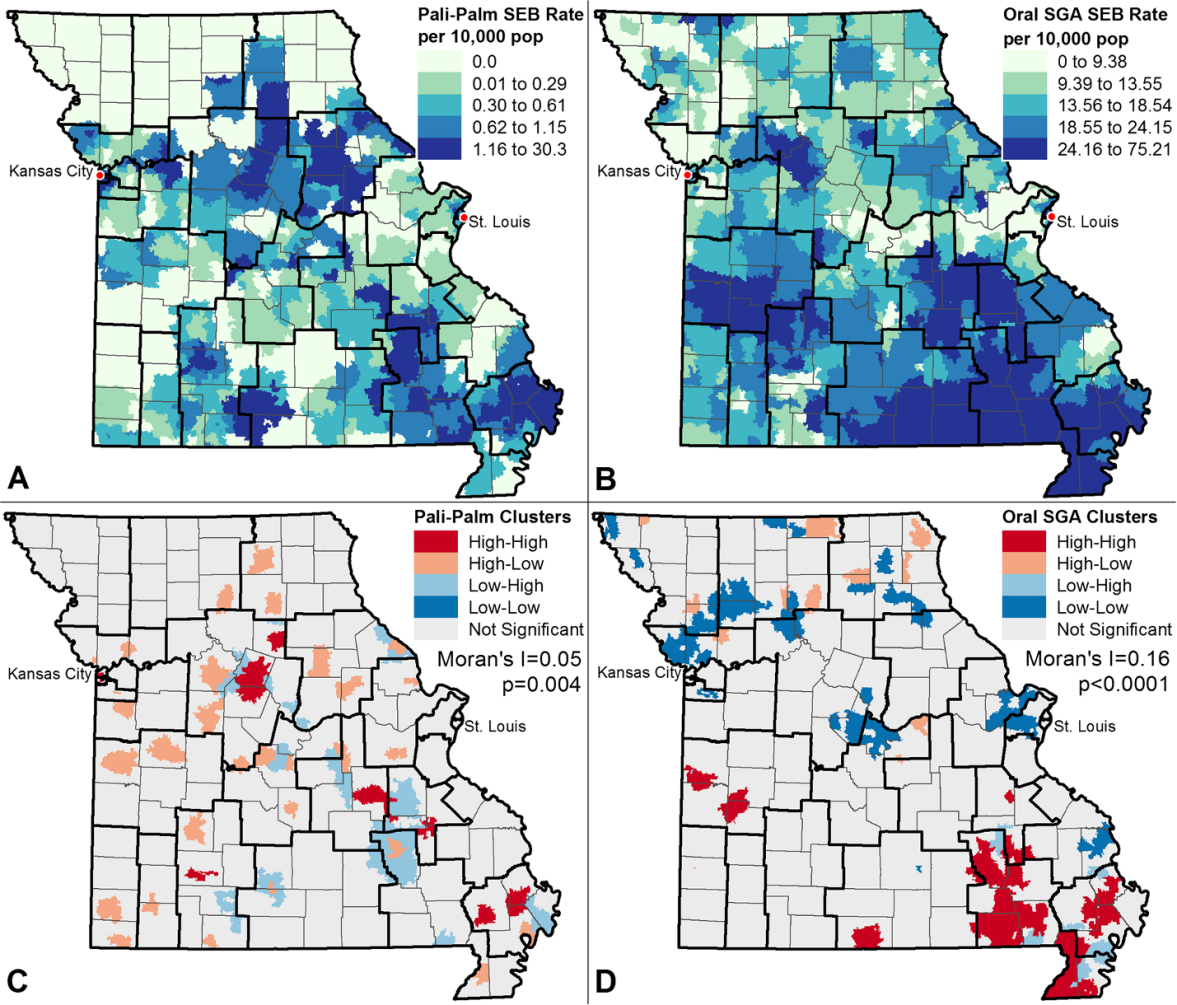
**Spatial Distribution of Drug Utilization Rates for A) Paliperidone Palmitate and B) Oral SGAs with Corresponding LISA Cluster Maps and Global Moran’s I Value for C) Paliperidone Palmitate and D) Oral SGAs.** Note: Maps **A** and **B** were developed using Spatial Empirical Bayes (SEB) smoothing methods. All maps were developed by aggregating data to zip codes. Dark black lines indicate the boundaries of the MHSAs and light grey lines indicate county boundaries.

**Figure 2:**
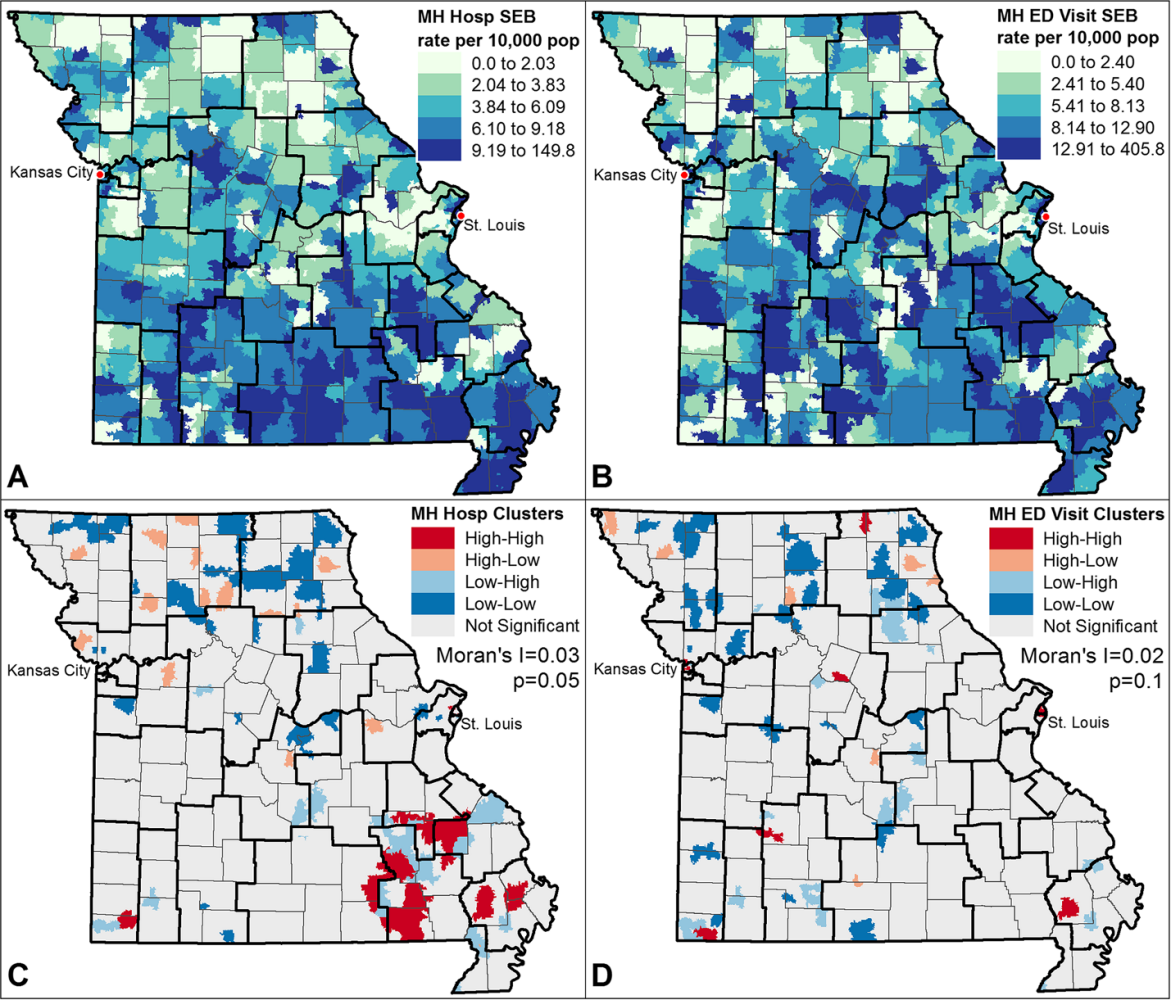
**Spatial Distribution of A) Primary Mental Health Hospitalizations and B) Primary Mental Health ED Visits with Corresponding LISA Cluster Maps and Global Moran’s I Value for C) Hospitalizations and D) ED Visits.** Note: Maps **A** and **B** were developed using Spatial Empirical Bayes (SEB) smoothing methods. All maps were developed by aggregating data to zip codes. Dark black lines indicate the boundaries of the MHSAs and light grey lines indicate county boundaries.

Figures 1 and 2 present results of the exploratory spatial data analyses for drug utilization rates (Figure 1) and hospitalization and ED visit rates (Figure 2). Figure 1A and B show the SEB smoothed rates for paliperidone palmitate and oral SGAs. Results indicate a clear spatial pattern of oral SGA use in the southern part of Missouri, which is predominantly rural with small towns and sparse population. Qualitatively, paliperidone palmitate utilization rates were higher in the southeast corner and north central portion of the state relative to other regions. Utilization of both drugs was higher in the major metropolitan areas of St. Louis and Kansas City. Figure 1C and D show the results of the Moran’s I and LISA analyses. The Moran’s I indicates that there is statistically significant, but weak, clustering of paliperidone palmitate (I = 0.05; p = 0.004) and a stronger clustering effect of oral SGAs (I = 0.16, p < 0.0001). The LISA maps indicate paliperidone palmitate had no strong visual pattern of local clustering (which is also reflected in the low Moran’s I of 0.05), but oral SGAs exhibited clusters of high use in the southeast corner, and a cluster of low utilization to the north of Kansas City and around St. Louis (which is reflected in a higher Moran’s I of 0.16).

Figure 2A and B show the SEB smoothed rates for ED visits and hospitalizations. Results indicate no clear spatial pattern for ED visits and hint at slightly higher hospitalizations in the southern portion of the state. The Moran’s I and LISA results strengthen this conclusion in Figure 2C and D. The Moran’s I is not statistically significant for ED visits, though LISA maps indicate the ED visits are high in the city cores of both Kansas City and St. Louis. The Moran’s I for hospitalizations is weak and borderline significant (I = 0.03; p = 0.05), and the LISA map indicates several larger clusters of high hospitalization in the southeast corner, and clusters of low rates in the northern region of the state.

Taken together, these results indicate that treatment exposure varied across regions, qualitatively indicated by visual interpretation of spatial patterns and quantitatively confirmed by statistically significant Moran’s I and LISA values, and that moderate spatial differences in the outcomes used to examine the effectiveness of paliperidone palmitate exist. The maps also qualitatively suggest both county-level and MHSA-level patterns exist for drug utilization.

**Figure 3:**
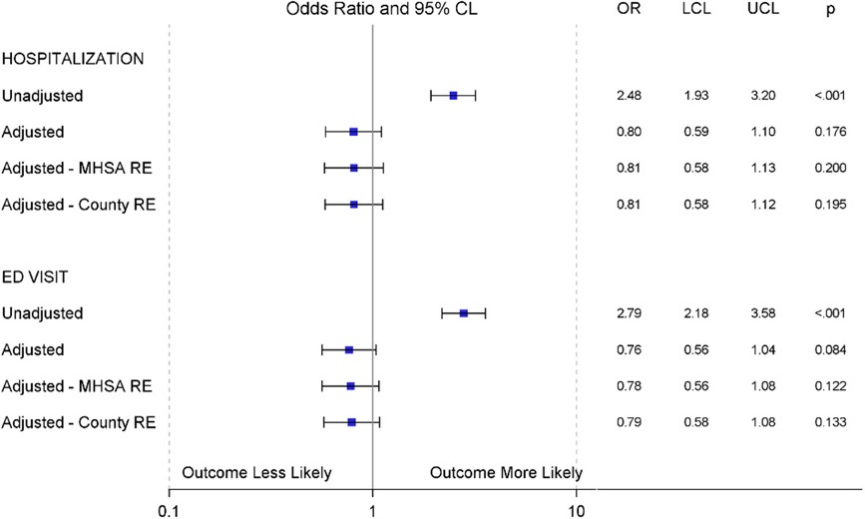
**Likelihood of mental-health related hospitalizations and emergency department visits after drug initiation.** Odds ratios compare patients starting paliperidone palmitate to patients starting oral second-generation antipsychotics. Adjusted models adjust for: cohort (paliperidone palmitate or oral SGA), patient demographics (age, sex, race), schizophrenia, baseline mental health and cardiometabolic co-morbidities, baseline psychotropic drug use, health care utilization (case management, outpatient visits), antipsychotic medication adherence, and frequency of mental-health hospitalizations or mental-health ED visits in the baseline period.

Additional file 1: Table S1 shows the baseline characteristics of patients starting paliperidone palmitate and oral SGAs. Figure 3 shows the results of the unadjusted and adjusted regression models for: a) no random effects (RE), b) MHSA-level random effects, and c) county-level random effects. After adjustment, the point estimates shifted in the direction of a protective benefit for persons initiating paliperidone palmitate compared to an oral SGA. The adjusted odds ratio (AOR) for hospitalizations was 0.80 (95% CI: 0.59-1.10) and for ED visits was 0.76 (95% CI: 0.56-1.04). Adding MHSA- or county-level random effects moved point estimates slightly toward the null, indicating that individual-level covariates were the most important source of potential bias in this case. The difference between MHSA- and county-level random effects on model results is negligible, suggesting either could be used as a level-2 unit in this analysis. Additional file 1: Tables S2 and S3 present results for these models as well as additional models which included slope-only and intercept and slope random effects.

**Figure 4:**
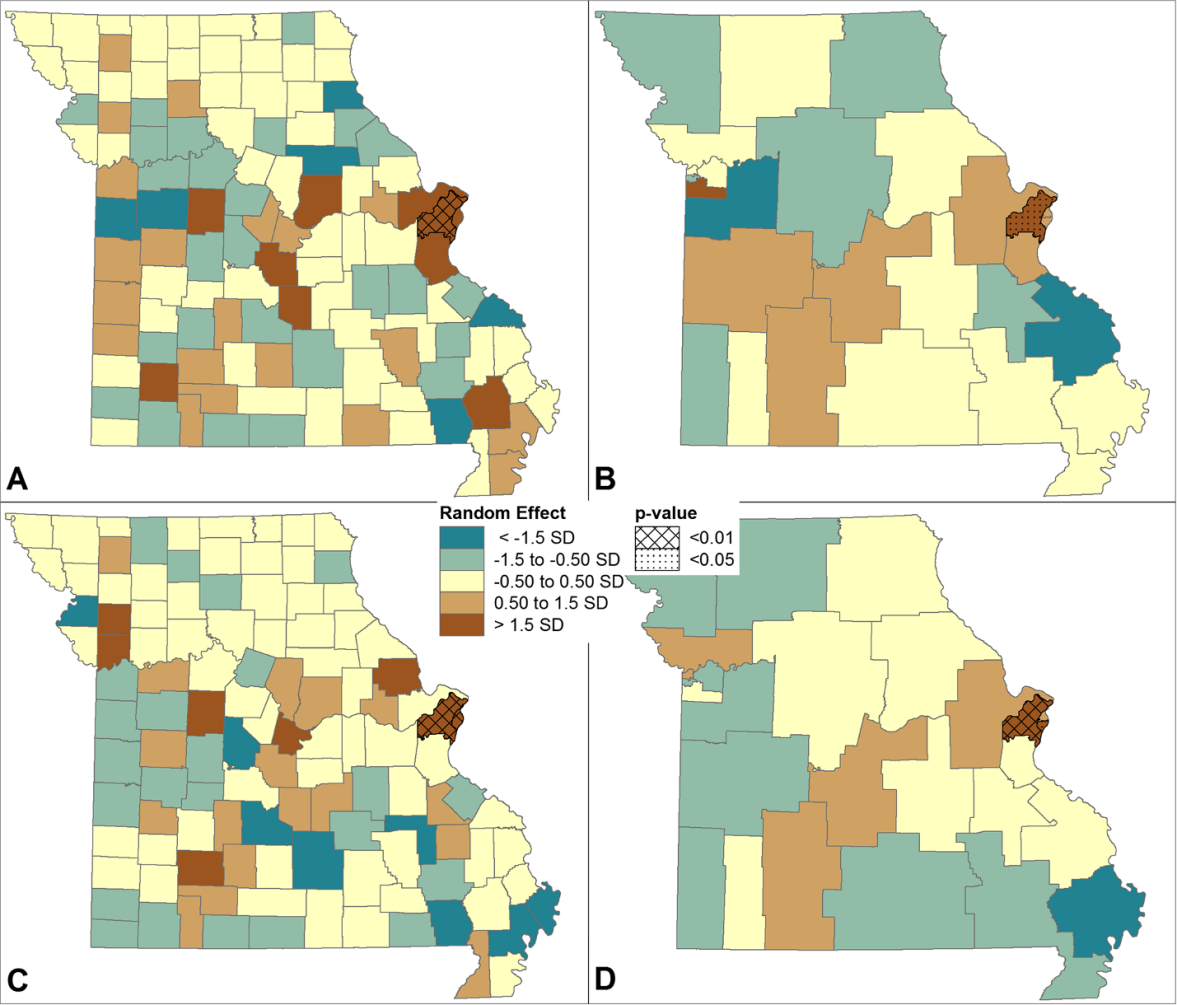
**Spatial Distribution of Random Effects for Adjusted Multivariate models for A) Primary Mental Health Hospitalizations for the county, B) Primary Mental Health Hospitalizations for the MHSA, C) Primary Mental Health ED Visits for the county and D) Primary Mental Health ED Visits for the MHSA.** Note: Values mapped are the estimated deviations from the global mean probability of a hospitalization or ED visit derived from the regression model. P-values indicate areas where the random effect was significantly different from the model mean. Values are classed using standard deviations in order to make them comparable across models.

Figure 4 maps the random effects, or deviations from the overall model mean value. P-values indicate if the random effect is significantly different from the mean. Figure 4A and B show county-level and MHSA-level random effects for mental health hospitalizations and indicate one county/MHSA just outside St. Louis where the likelihood of hospitalization is significantly higher than the study area as a whole. Figure 4C and D show random effects for mental health ED visits and indicate a high likelihood of a visit in the same region as well as in St. Louis.

Table 1 shows the variance components estimated from the multi-level models. Overall, about 1% of the variance in hospitalizations and 2% of the variance in ED visits is explained by county or MHSA differences. After adjusting for patient-level factors, this variance is approximately halved, suggesting that underlying population composition factors explain much of the variation across regions. However, since the random effects for adjusted models are statistically significant, there are still area-level contextual factors that are responsible for variation across regions.

**Table 1:** **Variance Components and Intra-class Correlation (ICC) for Random Effects Models**

	Var	SE	p-value	ICC
**Mental-health Related Hospitalizations**				
Unadjusted, MHSA Random Effect	0.034	0.017	<0.0001	1.0%
Adjusted, MHSA Random Effect	0.017	0.014	0.033	0.5%
Unadjusted, County Random Effect	0.044	0.020	<0.0001	1.3%
Adjusted, County Random Effect	0.023	0.018	0.029	0.7%
**Mental-health Related ED Visits**				
Unadjusted, MHSA Random Effect	0.071	0.027	<0.0001	2.1%
Adjusted, MHSA Random Effect	0.027	0.016	0.001	0.8%
Unadjusted, County Random Effect	0.070	0.023	<0.0001	2.1%
Adjusted, County Random Effect	0.037	0.019	0.001	1.1%

## Conclusions

Other studies have also suggested that geographic variation in treatments and outcomes can affect CER studies using observational data [7]. Our ESDA results indicated that geographic differences in treatment exposure (drug utilization) and outcomes (mental health hospitalizations and ED visits) are a potential confounder in this comparative effectiveness study of antipsychotics in the Missouri Medicaid system. Therefore, geography was incorporated into the multilevel models. The multilevel models indicated that adjusting for individual-level (compositional) factors explained about half the variation in outcomes evident across counties and MHSAs. This means that unmeasured compositional or additional contextual factors are possibly responsible for variation, for example as shown in the significant random effects in the St. Louis area. If there is sufficient sample, the recommended next step in a CER study would be to include area-level predictors (such as poverty rates or information on provider networks or practice patterns) in the level-2 portion of the multilevel model to better understand (and control for) the area-level contextual effects that drive these differences in outcomes. Due to our small sample size there was insufficient level-2 variance left after adding individual-level variables to continue with this analysis. In this case example, comparing the effectiveness of antipsychotic treatments, patient-level compositional factors were the most important driver of geographic variation. Similar results have been found in a variety of studies across public health disciplines; with many researchers agreeing that area-level characteristics only explain a modest amount of the variation in individual-level outcomes, perhaps in the order of 5-10% [37–39]. More research is needed to better estimate the range of confounding due to geography in observational comparative effectiveness studies and healthcare utilization outcomes. Exploratory spatial data analysis offers a suite of geographic tools that can be used to examine the potential confounding effect of geographical differences in the treatment and outcome being studied in observational CER studies. The results from ESDA can be used to examine whether geographic clustering conforms to specific geographic boundaries and can inform the choice of the level-2 unit for multilevel regression analyses. In the current study, the SEB rate maps indicated both county-level and MHSA-level patterns of hospitalization.

We suggest the PCORI and AHRQ guidelines could include ESDA and spatial statistical methods as additional techniques useful for statistical analyses of observational CER. In particular, the section describing descriptive statistics and unadjusted analyses could include a discussion of ESDA techniques and the section on adjusted analyses could discuss the use of area-level random effects and area-level predictors within multi-level model frameworks. The PCORI and AHRQ guidelines set high standards for the quality of CER studies and the inclusion of geographic methods will only serve to strengthen these guidelines. Not every study needs to adjust for geospatial variation, but we suggest it should be a CER best practice to test for possible geographic confounding. As most observational datasets commonly used for CER record some type of geographic indicator (e.g., zip code, county, address), conducting geographic analyses should become ubiquitous.

Beyond the utility and importance of the ESDA and spatial statistical analyses presented here, geographically explicit results can assist with the translation of research into practice. State and private health care delivery systems are often geographically defined. Policy makers seek to understand where people who need care are located and whether there are sufficient resources in a given region to support that population. Mapping results, as we do in this study, naturally informs the way in which policy makers intuitively examine the health system. They help to answer questions like: What resources need to be placed where? Which regions of the state have populations that might benefit from introduction of a new drug? Maps and geographic analyses provide valuable tools for hypothesis building and guiding policy decisions.

Study limitations should be acknowledged. First, although many covariates were included in the multivariable modeling, there may be omitted (or unmeasured) factors that are important to the comparisons being made. In addition, because this is an observational comparative effectiveness study in which treatment was not randomized, observed statistical *associations* do not necessarily mean there is a *causal* relationship. Therefore, effect estimates should be cautiously interpreted. With regard to this particular comparison, paliperidone palmitate was a newly launched drug at the time of the analysis and the sample size was limited which hindered our ability to find statistical significance. This is particularly problematic for mixed-effect models because small sample sizes within level-2 units lead to non-significant and uninformative random effects estimates. With a larger cohort, we could conduct additional model adjustment, such as propensity score matching or other restricted cohort methods, and then use multi-level models on the matched/restricted cohort. In addition, county- or MHSA-level fixed effects could be used to determine the contextual effects driving significant level-2 random effects. Second, this analysis provides a snapshot in time (one year of new starts) and selection bias and utilization rates may change over time as new drugs are adopted. Future analyses should consider multiple time points in order to examine how results of CER change over time as the new drug is incorporated in to the health system.

In summary, because geographic variation in health care delivery is ubiquitous, unmeasured confounding due to geography may be present in observational CER studies. Because measures of geographic location are commonly available in electronic medical records and administrative claims databases, we argue that CER best practices should include examination of possible geographic confounding in observational data. This paper illustrated methods for conducting such an examination.

## Electronic supplementary material

Additional file 1: **This file contains one supplementary figure and three supplementary tables for the manuscript.** The **Figure S1.** shows the inclusion/exclusion criteria for the study population, **Table S1.** shows the means and frequencies for characteristics of the study population, **Table S2.** shows the odds ratios and 95% confidence intervals for the models displayed in Figure 3, and **Table S3.** shows additional model results. (PDF 373 KB)

## Abbreviations

AHRQ: Agency for Healthcare Research and Quality
CER: Comparative Effectiveness Research
ED: Emergency department
ESDA: Exploratory spatial data analysis
LISA: Local indicator of spatial association
MHSA: Mental Health Service Area
PCORI: Patient Centered Outcomes Research Institute
PCOR: Patient Centered Outcomes Research
PP: Paliperiodone palmitate
SEB: Spatial empirical Bayes
SES: Socio-economic status
SGA: Second-generation antipsychotic.

## References

[1] Velentgas P, Dreyer N, Nourjah P, Smith S, Torchia M (2013). Developing a Protocol for Observational Comparative Effectiveness Research: A User's Guide. AHRQ Publication No 12(13)-EHC099.

[2] Patentint Centered Outcomes Research Institute (PCORI) (2012). Public comment draft report of the Patient-Centered Outcomes ResearchInstitute (PCORI) Methodology Committee.

[3] Folland S, Stano M (1989). Sources of small area variations in the use of medical care. J Health Econ.

[4] Wennberg J, Gittelsohn A (1982). Variations in medical care among small areas. Sci Am.

[5] Kawachi I, Berkman LF (2003). Neighborhoods and health.

[6] Ricketts R, Savitz L, Gesler W (1994). Geographic Methods in Health Services Research.

[7] Song Y, Skinner J, Bynum J, Sutherland J, Wennberg JE, Fisher ES (2010). Regional variations in diagnostic practices. N Engl J Med.

[8] Dartmouth Medical School. Center for the Evaluative Clinical Sciences, American Hospital Association, Center for Health Care Leadership (1996). The Dartmouth atlas of health care.

[9] Dartmouth Medical School, Center for the Evaluative Clinical Sciences, Maine Medical Center, Center for Outcomes Research and Evaluation (1999). The Dartmouth atlas of cardiovascular health care.

[10] Dartmouth Medical School. Center for the Evaluative Clinical Sciences., Maine Medical Center, Center for Outcomes Research and Evaluation (2000). The Dartmouth atlas of musculoskeletal health care.

[11] Pickle L, Mungiole M, Jones G, White A (1996). Atlas of United States mortality.

[12] Diez Roux AV (2001). Investigating neighborhood and area effects on health. Am J Public Health.

[13] Cooper MM (1996). The Dartmouth Atlas of Health Care: what is it telling us?. Health Syst Rev.

[14] Fang G, Brooks JM, Chrischilles EA (2010). A new method to isolate local-area practice styles in prescription use as the basis for instrumental variables in comparative effectiveness research. Med Care.

[15] Basu A (2011). Estimating Decision-Relevant Comparative Effects Using Instrumental Variables. Stat Biosci.

[16] Brookhart MA, Rassen JA, Wang PS, Dormuth C, Mogun H, Schneeweiss S (2007). Evaluating the validity of an instrumental variable study of neuroleptics: can between-physician differences in prescribing patterns be used to estimate treatment effects?. Med Care.

[17] Phelan JC, Link BG, Tehranifar P (2010). Social conditions as fundamental causes of health inequalities: theory, evidence, and policy implications. J Health Soc Behav.

[18] Sampson R, Morenoff J, Gannon-Rowley T (2002). Assessing "neighborhood effects": Social processes and new directions in research. Annu Rev Sociol.

[19] Oakes J, Rossi P (2003). The measurement of SES in health research: current practice and steps toward a new approach. Soc Sci Med.

[20] Berkman L (1995). Role of Social-relations in health promotion. Psychosom Med.

[21] Berkman L, Glass T, Brissette I, Seeman T (2000). From social integration to health: Durkheim in the new millennium. Soc Sci Med.

[22] Diez Roux AV (2008). Next steps in understanding the multilevel determinants of health. J Epidemiol Community Health.

[23] Cubbin C, Winkleby MA (2005). Protective and harmful effects of neighborhood-level deprivation on individual-level health knowledge, behavior changes, and risk of coronary heart disease. Am J Epidemiol.

[24] Winkleby M, Cubbin C, Ahn D (2006). Effect of cross-level interaction between individual and neighborhood socioeconomic status on adult mortality rates. Am J Public Health.

[25] Taylor CB, Ahn D, Winkleby MA (2006). Neighborhood and individual socioeconomic determinants of hospitalization. Am J Prev Med.

[26] Claudio L, Tulton L, Doucette J, Landrigan PJ (1999). Socioeconomic factors and asthma hospitalization rates in New York City. J Asthma.

[27] Agency for Healthcare Research and Quality (AHRQ): **Clinical Classifications Software for Mental Health and Substance Abuse (CCS-MHSA).** [http://www.hcup-us.ahrq.gov/toolssoftware/mhsa/mhsa.jsp]

[28] Morrato EH, Druss B, Hartung DM, Valuck RJ, Allen R, Campagna E, Newcomer JW (2010). Metabolic testing rates in 3 state Medicaid programs after FDA warnings and ADA/APA recommendations for second-generation antipsychotic drugs. Arch Gen Psychiatry.

[29] Morrato EH, Druss BG, Hartung DM, Valuck RJ, Thomas D, Allen R, Campagna E, Newcomer JW (2011). Small area variation and geographic and patient-specific determinants of metabolic testing in antipsychotic users. Pharmacoepidemiol Drug Saf.

[30] Anselin L, Kim Y, Syabri I (2004). Web-based analytical tools for the exploration of spatial data. J Geogr Syst.

[31] Anselin L (2006). How (not) to lie with spatial statistics. Am J Prev Med.

[32] Bailey TC, Gatrell AC (1995). Interactive spatial data analysis.

[33] Anselin L, Florax RJGM, Rey SJ (2004). Advances in spatial econometrics: methodology, tools and applications.

[34] Anselin L (1995). Local Indicators of Spaital Association - LISA. Geogr Anal.

[35] Gelman A, Hill J (2006). Data Analysis Using Regression and Multilevel/Hierarchical Models.

[36] Diez-Roux AV (2000). Multilevel analysis in public health research. Annu Rev Public Health.

[37] Leventhal T, Brooks-Gunn J (2000). The neighborhoods they live in: The effects of neighborhood residence on child and adolescent outcomes. Psychol Bull.

[38] Riva M, Gauvin L, Barnett TA (2007). Toward the next generation of research into small area effects on health: a synthesis of multilevel investigations published since July 1998. J Epidemiol Community Health.

[39] Chaix B, Rosvall M, Merlo J (2007). Recent increase of neighborhood socioeconomic effects on ischemic heart disease mortality: A multilevel survival analysis of two large Swedish cohorts. Am J Epidemiol.

[CR40] The pre-publication history for this paper can be accessed here:http://www.biomedcentral.com/1472-6963/14/355/prepub

