# Supplementary material for: Adjusting for geographic variation in observational comparative effectiveness studies: a case study of antipsychotics using state Medicaid data
Source: BMC Health Serv Res. 2014 Aug 27;14:355. doi: 10.1186/1472-6963-14-355 (PMC4161848; doi:10.1186/1472-6963-14-355)
Supplement: Supplementary file 1 — Additional file 1: This file contains one supplementary figure and three supplementary tables for the manuscript. The Figure S1. shows the inclusion/exclusion criteria for the study population, Table S1. shows the means and frequencies for characteristics of the study population, Table S2. shows the odds ratios and 95% confidence intervals for the models displayed in Figure 3, and Table S3. shows additional model results. (PDF 373 KB) [file 12913_2013_3464_MOESM1_ESM.pdf]

Supplementary Figure 1. Study population selection

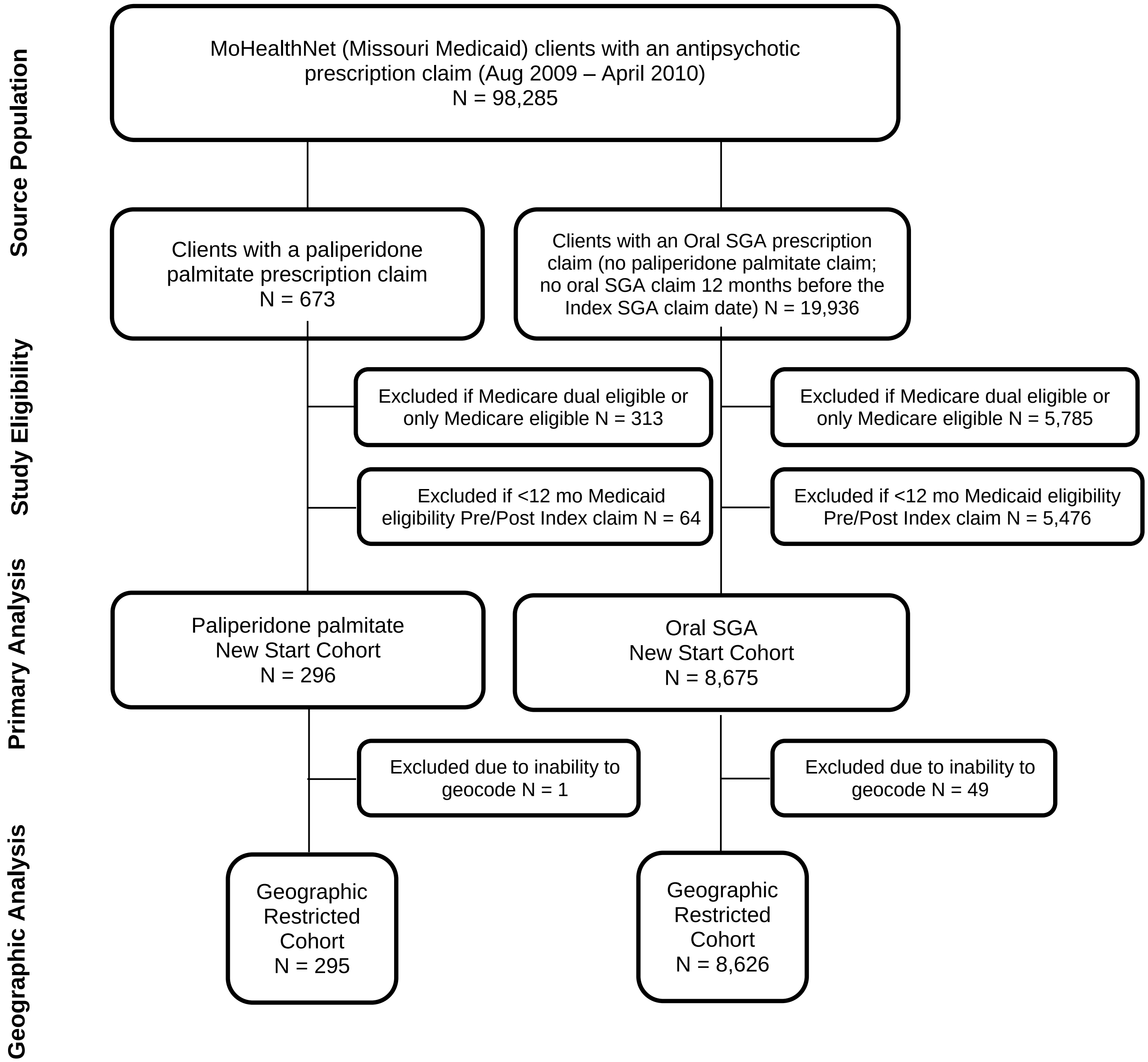

Supplementary Table 1. Characteristics of patients in the new start cohort

Selected baseline characteristics	Paliperidone Palmitate N=296		Oral SGA N = 8,675		Test Statistic	Degree(s) of Freedom	p-value
	mean	sd	mean	sd			
**Patient age (**at index prescription, yr)	36.6	13.0	25.6	15.8	14.23	325.51	<0.001
**Patient demographics**	%	n	%	n			
Male	50.0	148	42.7	3,708	6.15	1	0.01
Race White	56.1	166	78.1	6,779	88.36	2	<0.001
Black/African American	41.6	123	19.3	1,678			
Other/Unknown	2.4	7	2.5	218			
Residence in an urban county	74.7	221	65.3	5,648	11.03	1	<0.001
**Mental Health and Substance Abuse Classifications**							
Schizophrenia and other psychotic disorders	81.8	242	11.7	1,015	1165.96	1	<0.001
Mood disorders	70.6	209	71.5	6,201	0.11	1	0.74
Anxiety disorders	47.3	140	44.2	3,833	1.12	1	0.29
Substance-related disorders	37.5	111	17.8	1,542	74.09	1	<0.001
Alcohol-related disorders	20.9	62	9.1	790	46.68	1	<0.001
Attention-deficit, conduct, disruptive behavior disorders	19.6	58	34.3	2,972	27.52	1	<0.001
Personality disorders	18.6	55	7.1	620	53.78	1	<0.001
Suicide and intentional self-inflicted injury	17.2	51	9.6	835	18.60	1	<0.001
Developmental disorders	15.9	47	7.9	684	24.44	1	<0.001
Delirium, dementia, and other cognitive disorders	5.1	15	2.8	243	5.26	1	0.02
Adjustment disorders	3.4	10	10.4	899	15.34	1	<0.001
Disorders diagnosed in infancy, childhood, adolescence	3.4	10	5.9	516	3.42	1	0.06
Impulse control disorders, not elsewhere classified	2.7	8	5.3	456	3.81	1	0.05
Number of unique classifications							
0	1.0	3	5.1	444	88.17	2	<0.001
1 -2	33.8	100	56.4	4,896			
3 or more	65.2	193	38.4	3,335			
**Psychotropic Use (past 12 months)**							
Other antipsychotic not included in the index cohort	91.6	271	3.6	314	3630.86	1	<0.001
Mood Stabilizer	48.6	144	18.2	1,580	170.79	1	<0.001
Antidepressant	59.5	176	51.8	4,491	6.78	1	0.01
Benzodiazapine	45.3	134	27.9	2,417	42.63	1	<0.001
**General Health Care Utilization**							
Received care at a CMHC	81.8	242	34.9	3,031	270.74	1	<0.001
Received case management	64.9	192	13.8	1,201	568.05	1	<0.001

SGA indicates second-generation antipsychotic; CMHC, Community Mental Health Center.
Baseline characteristics are defined based on claims during 12 months preceding the Index claim date.
Residence in an urban county excludes patients with a non-Missouri county of residence, n=31 (all in OSGA cohort)
Test statistic is from Welch’s t-test for patient age and Pearson’s chi-square test of association for categorical variables.

Supplementary Table 2. Multivariate Regression Model Results, Odds Ratios and 95% Confidence Intervals

	Random Slope and Intercept Model		Random Intercept only Model		Random Slope Only Model	
	OR (95% CI)†	p-value	OR (95% CI)†	p-value	OR (95% CI)†	p-value
**Hospitalization**						
Unadjusted Models	2.5 (1.9-3.2)	<0.0001	2.5 (1.9-3.2)	<.0001	2.5 (1.9-3.2)	<.0001
w/MHSA Random Effect	2.6 (1.9-3.5)	<0.0001	2.4 (1.8-3.2)	<.0001	2.6 (1.9-3.5)	<0.0001
w/County Random Effect	2.9 (2.2-3.9)	<0.0001	2.4 (1.8-3.1)	<.0001	2.9 (2.2-3.9)	<0.0001
Adjusted Models*	0.8 (0.6-1.1)	0.1759	0.8 (0.6-1.1)	0.1759	0.8 (0.6-1.1)	0.1759
w/MHSA Random Effect	0.8 (0.6-1.2)	0.3149	0.8 (0.6-1.1)	0.2002	0.8 (0.6-1.2)	0.3149
w/County Random Effect	0.9 (0.6-1.3)	0.5636	0.8 (0.6-1.1)	0.1947	0.9 (0.6-1.3)	0.5636
**ED Visit**						
Unadjusted Models	2.8 (2.2-3.6)	<0.0001	2.8 (2.2-3.6)	<.0001	2.8 (2.2-3.6)	<0.0001
w/MHSA Random Effect	2.8 (2.1-3.7)	<0.0001	2.7 (2.1-3.5)	<.0001	2.9 (2.1-3.9)	<0.0001
w/County Random Effect	3.2 (2.4-4.3)	<0.0001	2.7 (2.1-3.5)	<.0001	3.2 (2.4-4.3)	<0.0001
Adjusted Models*	0.8 (0.6-1.0)	0.0844	0.8 (0.6-1.0)	0.0844	0.8 (0.6-1.0)	0.0844
w/MHSA Random Effect	0.8 (0.6-1.1)	0.1335	0.8 (0.6-1.1)	0.1221	0.8 (0.6-1.1)	0.1953
w/County Random Effect	0.8 (0.6-1.2)	0.3203	0.8 (0.6-1.1)	0.1318	0.8 (0.6-1.2)	0.3203

† Odds ratios show the odds of outcome (mental health hospitalization or ED visit) for patients treated with paliperidone palmitate vs. oral SGAs.
* Models were adjusted for cohort (paliperidone palmitate or oral SGA), patient demographics (age, sex, race), urbanicity, schizophrenia, baseline mental health and cardiometabolic co-morbidites, baseline psychotropic drug use, health care utilization (case management, outpatient visits), antipsychotic medication adherence, and frequency of mental-health hospitalizations and mental-health ED visits in the baseline period.

Supplementary Table 3. Multivariate Regression Model Results, Variance Components with Standard Errors for Random Effects Models

	Random Slope and Intercept Model				Random Intercept only Model		Random Slope Only Model	
	Variance (SE) Intercept	p-value	Variance (SE) Slope†	p-value	Variance (SE)	p-value	Variance (SE)	p-value
**Hospitalization**								
Unadjusted Models								
w/MHSA Random Effect	0.022 (0.036)	0.124	0.049 (0.042)	1	0.034 (0.017)	<0.0001	0.043 (0.020)	<0.0001
w/County Random Effect	0.004 (0.031)	0.252	0.023 (0.033)	1	0.017 (0.014)	0.033	0.024 (0.017)	0.0102
Adjusted Models*								
w/MHSA Random Effect	0.073 (0.024)	1	0 (0)	1	0.044 (0.020)	<0.0001	0.054 (0.023)	<0.0001
w/County Random Effect	0.042 (0.020)	1	0 (0)	1	0.023 (0.018)	0.029	0.031 (0.021)	0.0096
**ED Visit**								
Unadjusted Models								
w/MHSA Random Effect	0.043 (0.020)	1	0 (0)	1	0.071 (0.027)	<0.0001	0.068 (0.025)	<0.0001
w/County Random Effect	0.024 (0.017)	1	0 (0)	1	0.027 (0.016)	0.001	0.026 (0.015)	0.0016
Adjusted Models*								
w/MHSA Random Effect	0.054 (0.023)	1	0 (0)	1	0.070 (0.023)	<0.0001	0.073 (0.024)	<0.0001
w/County Random Effect	0.031 (0.021)	1	0 (0)	1	0.037 (0.019)	0.000	0.042 (0.020)	<0.0001

* Models were adjusted for cohort (paliperidone palmitate or oral SGA), patient demographics (age, sex, race), urbanicity, schizophrenia, baseline mental health and cardiometabolic co-morbidites, baseline psychotropic drug use, health care utilization (case management, outpatient visits), antipsychotic medication adherence, and frequency of mental-health hospitalizations and mental-health ED visits in the baseline period.

† Variance of slope was estimated at 0 for most models.
